# Fatal child abuse detected by systematic post-mortem fundus photograph in sudden death in infancy

**DOI:** 10.1007/s00414-024-03220-4

**Published:** 2024-04-23

**Authors:** Jean-Baptiste Ducloyer, Frédérique Jossic, Valentine VAN Goethem, David Lebosse, Charlène Cornee, Guylène Le Meur, Mathilde Ducloyer

**Affiliations:** 1 CHU Nantes, service d’ophtalmologie, Nantes Université, 1 place Alexis Ricordeau, Nantes, 44093 France; 2 Institut d’histopathologie (IHP Group), Angers, France; 3 CHU Nantes, service de médecine légale, Nantes Université, Nantes, France; 4 CHU Nantes, INSERM, Nantes Université, Nantes, CIC 1413, 44000 CIC France

**Keywords:** Sudden unexpected death in infancy, SUDI, Post-mortem, Retinal haemorrhages, Abusive head trauma, RetCam

## Abstract

In living children, the use of a wide field fundus camera such as RetCam is the gold standard practice to document retinal haemorrhages in suspected cases of abusive head trauma (AHT). In case of sudden unexpected death in infancy (SUDI), child abuse must be considered as a possible cause of death and an eye examination is required. However, no example of post-mortem fundus photograph (PMFP) of retinal haemorrhages related to AHT is yet available for clinicians.

We report a SUDI case, with no external traumatic lesions or limb fractures, for which prompt PMFP showed retinal haemorrhages typical of AHT: child abuse was subsequently confirmed by the forensic investigation. We discuss why PMFP is a relevant screening test to detect retinal haemorrhages in the case of SUDI and why the use of the RetCam should be further investigated.

## Introduction

Sudden death in infancy (SUDI) is defined as an abrupt and unexpected death before one year of age. It is the first cause of death after the neonatal period in France and concerns 35 infants per 100 000 live births every year in Europe [1]. When clinicians are confronted with SUDI, they must meticulously research a cause of death before concluding sudden infant death syndrome (SIDS) [2, 3]. Abusive head trauma (AHT) is a particularly complicated cause of SUDI because it is hardly conceivable, difficult to assert, and because its identification will result in a judicial enquiry for homicide [4, 5]. As retinal haemorrhages (RH) are a key sign for AHT (sensitivity = 75% and specificity = 94%) [6], eye examination and fundus documentation are essential. In living children, a wide field fundus camera such as the RetCam (Clarity Medical Systems USA) is the gold standard to acquire retinal images in suspected cases of child abuse [7].

However, no post-mortem fundus photograph (PMFP) showing RH related to AHT has ever been published. Therefore, clinicians examining the fundus in SUDI cases may be unaware of the expected appearance of RH after death. Here we report the case of a deceased child, admitted at the hospital for SUDI, with no external traumatic lesions or limb fractures, for which prompt RetCam PMFP showed RH typical of AHT. Child abuse was subsequently confirmed by a forensic investigation which included computed tomography, autopsy and pathological examination. We discuss why PMFP is a relevant screening test to detect RH in the case of SUDI and why the use of the RetCam should be further investigated for this test.

## Case description

A six-week-old boy was managed by the mobile emergency service for a cardio-respiratory arrest. The child was born at term and did not present health problems during pregnancy or after birth. The medical history of the child just before the death was unclear though a malaise was suggested by the close relative. The period between the first symptoms and the call for medical advice was more than an hour. The resuscitation was limited (chest compression, mask ventilation). He was transported to the hospital for post-mortem investigations, including a complete clinical examination, PMFP, biological sample collection and post-mortem imaging.

**Fig. 1:**
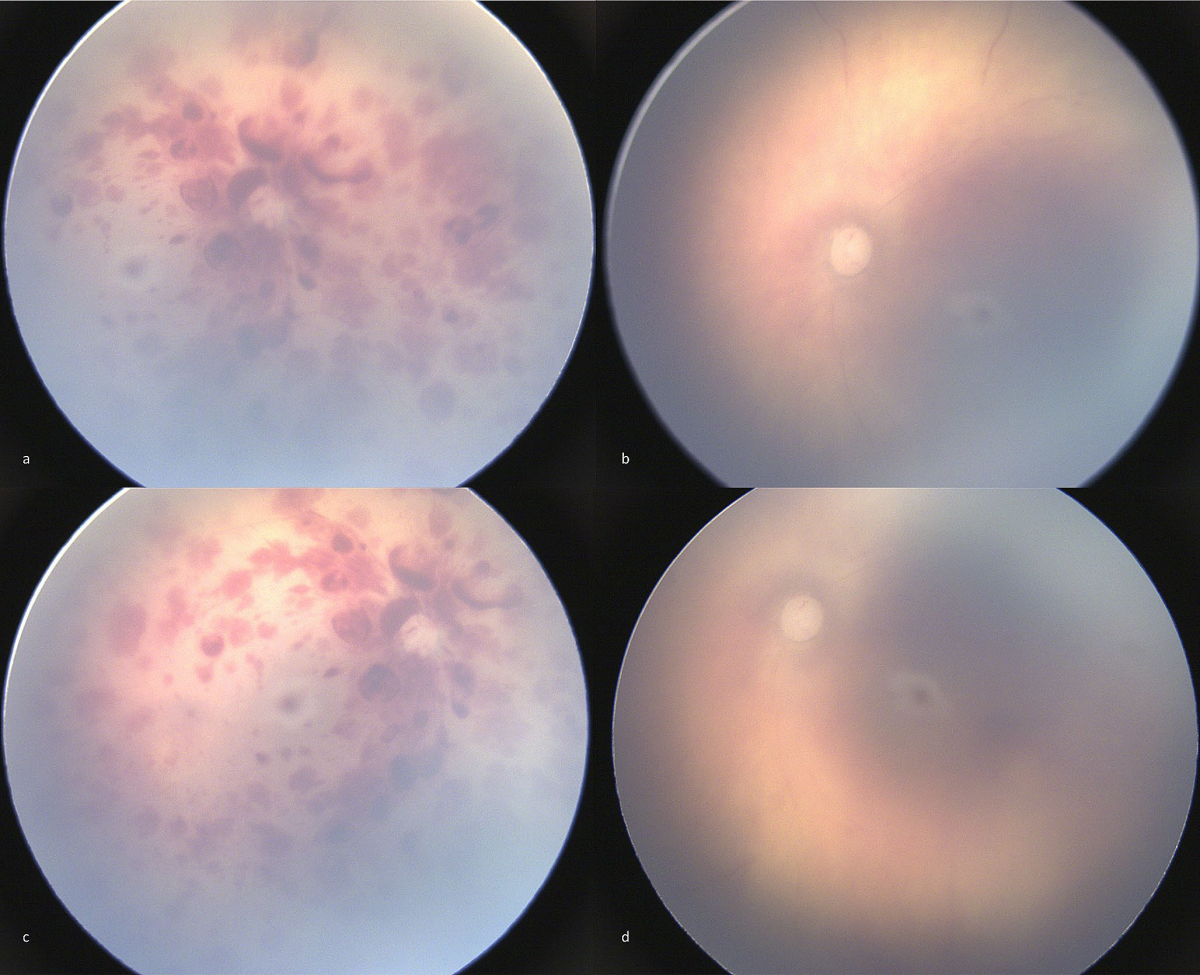
Post-mortem fundus photographs. On the right eye (**a**, **c**), retinal haemorrhages are multilayered and extensive from the posterior pole to the periphery. On the left eye (**b**, **d**), there is no visible retinal haemorrhage on the posterior pole and mid periphery. Post-mortem interval between death and examination was approximately 2 h

Clinical examination was performed 1,5 h after the first call to the emergency services. The child weighed 4.205 kg and measured 58 cm. The cranial perimeter was measured at 38 cm. The examination of the skin did not show any traumatic injury. Cadaveric signs were: no diffuse rigor mortis, posterior livor mortis and a temperature at 30°. The eye fundus was examined with the RetCam and PMFP were performed (Fig. 1). On the right eye RH were found that were too numerous to count, multilayered and extensive from the posterior pole to the periphery. On the left eye, only a few discrete RH were visible on the extreme periphery and no RH were visible on the posterior pole nor mid periphery. The suspicion of fatal child abuse was raised at this time which in turn directed other forensic investigations and alerted the court system.

A whole-body post-mortem computed tomography (PMCT) and X ray of the entire skeleton were performed at approximately 6 h after death. PMCT showed a right subdural hematoma, a subarachnoid haemorrhage in the right sylvian fissure and an epidural hematoma (Fig. 1). No fracture was detected on the X ray nor on the PMCT.

Autopsy was performed two days after death. This revealed a haemorrhagic infiltration of the posterior cervical muscles, as well as a right subdural hemispheric hematoma with rupture of the homolateral bridging veins. This was associated with a diffuse subarachnoid haemorrhage, in particular surrounding the brainstem. Gross examination of the other organs did not show signs of congenital malformation or other macroscopic abnormalities. Toxicology screening was negative. The cerebrospinal fluid collected by a lumbar puncture was considered to be haemorrhagic, confirmed by the cytology, and remained sterile after culture. Blood culture showed the presence of several common bacteria likely to result from skin contamination. Virological analyses (pharyngeal-nasal swab for Influenza, Sars-CoV2) were negative; other biological samples (blood formula, CRP, PCT) were normal.

Pathological examination showed the presence of recent multifocal subarachnoid haemorrhages of the cervical spine, the presence of a recent subdural hematoma and a right parietal subarachnoid haemorrhage. It did not reveal any congenital malformation or any preexisting disease. The pathological examination of the right eye showed extended haemorrhages of the nerve fiber layer, internal nuclear layer and focally reaching the outer nuclear layer. A haemorrhage of the optic nerve head and an intrascleral haemorrhage was also detected (Fig. 3).

The overall conclusion of the post mortem investigations was that the child was deceased as a result of a shaken baby syndrome.

**Fig. 2:**
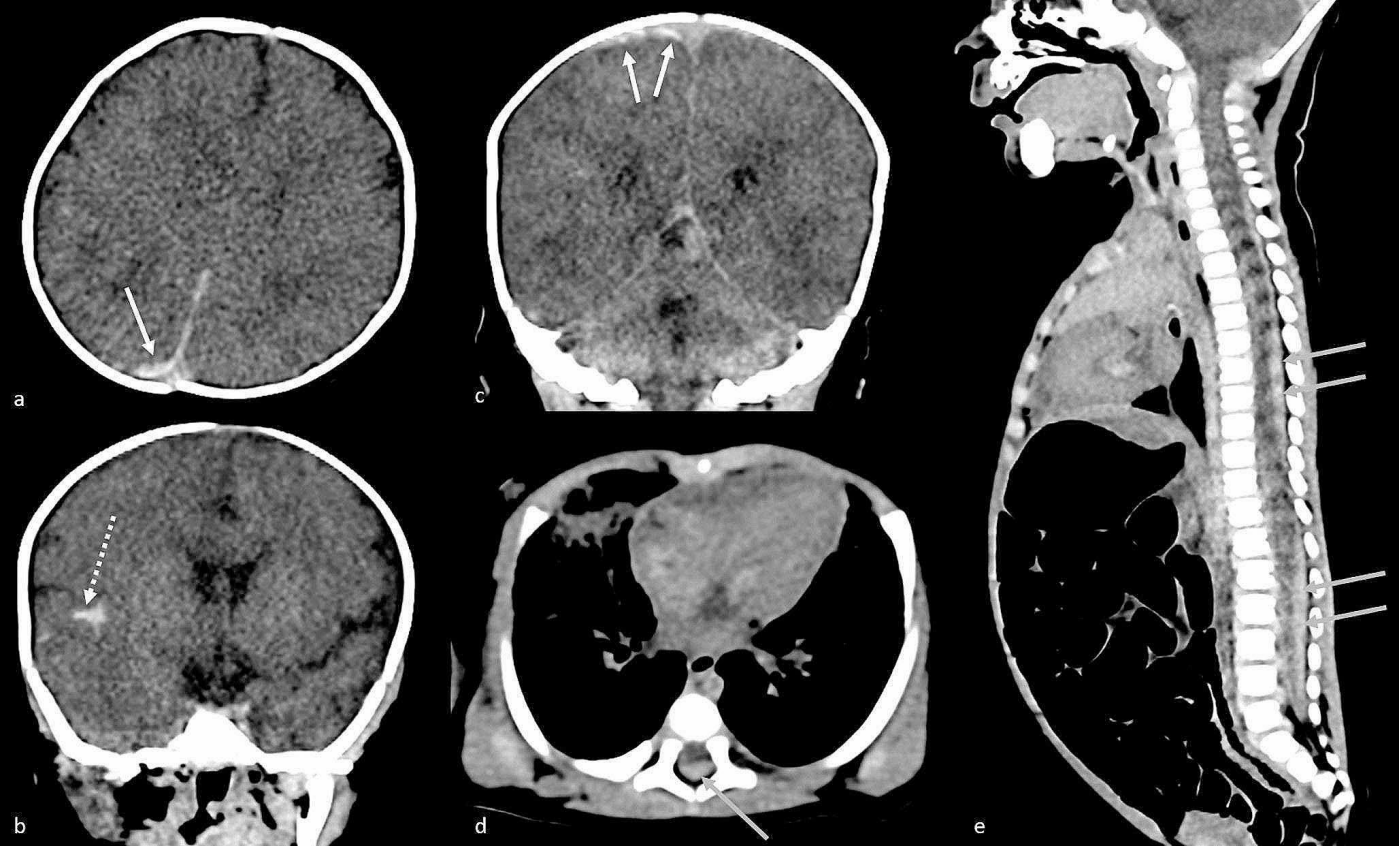
Post mortem computed tomography. Soft tissue reconstruction. On the axial view of the brain (**a**), subdural hematoma is visible as a hyperdensity of the subdural space of the parietal convexity (arrow). On the coronal view of the brain (**b**, **c**), subarachnoid haemorrhage is visible as a spontaneous hyperdensity of the sylvian fissure (dotted arrow) and temporal subdural hematoma is visible as a lens-like hyperdensity of the subdural space (arrowhead). On the axial (**d**) and sagittal view (**e**) of the spinal cord, epidural hematoma is visible as a spontaneous epidural hyperdensity around the spinal cord (gray arrows)

**Fig. 3:**
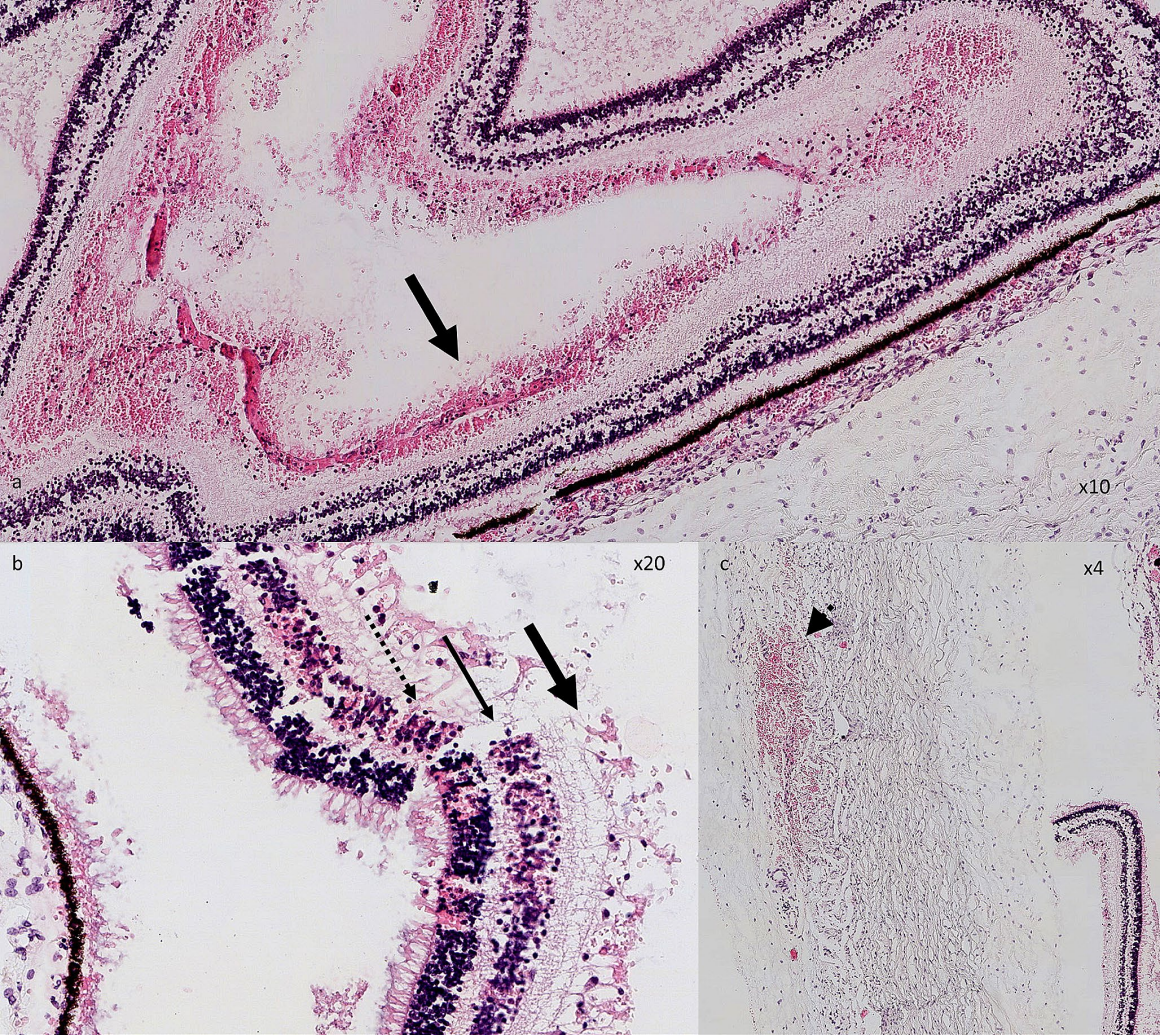
Pathological eye examination of the right eye. Multilayered extended retinal haemorrhages are visible in the retinal fiber layer (black arrows; **a**, magnification x2 and b, magnification x20), internal nuclear layer (thin arrow; **b**) and in the outer retinal layer (dotted arrow; **b**). Intrascleral haemorrhage is also visible (arrowhead; **c**, magnification x4)

## Discussion

In case of SUDI, fatal child abuse must be considered and eye examination is systematically required, especially, as in this case, shaken baby syndrome does not necessarily imply the presence of visible external traumatic lesions or limb fractures. Although fundus photography is the gold standard in living children, pathological examination is the reference for screening for RH in deceased children: the American Academy of Pediatrics recommends post-mortem eye removal for pathological examination in the case of SUDI that has not clearly resulted from a witnessed severe accidental head trauma or readily diagnosed systemic medical conditions [8]. Indeed, only a few post-mortem fundus examinations have been described in deceased children and these cases have a number of limitations. In particular, classic indirect ophthalmoscopy does not allow image acquisition while indirect ophthalmoscopy associated with a smartphone is challenging and requires specific training [9]. While endoscopy bypasses post-mortem opacification of cornea and lens it is invasive and it requires training and a specific endoscope [10, 11].

In 2023, Ducloyer et al. described for the first time the technique and advantages of RetCam PMFP in deceased children [12]. It allows the assessment of a larger area of retina than with indirect ophthalmoscopy [13]. The interpretation is immediate, so the detection of RH as soon as the child has arrived at the hospital can promptly alert the clinicians and the justice system, as in the case described here. As it is non-invasive, it does not raise issues of eye removal which includes the problem of consent from family and caregivers, the need for a trained pathologist which can delay autopsy results, and the risk of enucleation artifacts [14–16]. Moreover, PMFP will not jeopardize eye removal for the pathological examination which would be required in the case of RH or if the image quality is insufficient. Indeed, Ducloyer et al. found that the quality of the images was not sufficient to state presence or absence of RH in 17% of cases, mainly when PMFP was performed more than 18 h after death [12]. The other limitation of PMFP is the impossibility to document histological specific findings such as optic nerve sheath/intrascleral/extraocular muscles/orbital fat haemorrhages, age estimation of the bleeding and the probability of bleedings at more than one time point [14].

In summary, this is the first case report in which the appearance of RH related to AHT is shown with fundus photographs taken after death. Furthermore, it highlights the many advantages of the RetCam PMFP as a screening test when performed as soon as a SUDI case arrives at the hospital. In SUDI cases, systematic eye examination is required as signs of trauma may not be evident in the context of the death, assessment of the death scene and external examination of the body. Pathological examination of the eye remains the gold standard to document any suspicion of child abuse and further studies are needed to assess under what conditions the absence of RH on PMFP of sufficient quality could avoid the removal of ocular and orbital tissue, which is difficult and traumatic.

## Abbreviations

AHT: abusive head trauma
OMIN: observatoire national des morts inattendues du nourrisson
PMFP: post-mortem fundus photograph
RH: retinal haemorrhages
SIDS: sudden infant death syndrome
SUDI: sudden unexpected death in infancy
